# Success Rate Comparis on of External Dacryocystorhinostomy with 5-FU Application or Silicone Tube Intubation

**DOI:** 10.1155/2022/2941283

**Published:** 2022-07-07

**Authors:** Ayşe Burcu Dirim, Ibrahim Çağrı Türker, Ceylan Uslu Doğan, Selam Yekta Şendül, Emine Betül Akbaş Özyürek

**Affiliations:** University of Health Sciences, Sisli Hamidiye Etfal Training and Research Hospital, Department of Ophthalmology, Istanbul, Turkey

## Abstract

**Aims:**

To compare anatomical and functional success rates in patients with primary acquired nasolacrimal duct obstruction undergoing external dacryocystorhinostomy (EX-DCR) either with adjunctive 5-fluorouracil (5-FU) or silicone tube intubation.

**Methods:**

In this retrospective comparative study, 37 eyes in 32 patients who underwent EX-DCR with adjunctive 5-FU (5-FU group) and 43 eyes in 40 patients who underwent EX-DCR with silicone intubation (controls) between 2018 and 2019 were included.

**Results:**

The mean age of patients in 5-FU and control groups was 59.8 ± 9.4 and 57.0 ± 15.3 years, respectively. The mean follow-up was 18.70 ± 3.47 months in the 5-FU group and 21.38 ± 7.76 months in the control group. Anatomical success was determined based on patency rates at the time of irrigation and recurrence, while subjective symptoms (improvement in tearing) were used to evaluate the functional success. Lacrimal patency rates in 5-FU and control groups were 83.3% and 86.0%, respectively, while recurrence was observed in 16.2% of 5-FU and 14.0% of control subjects. The two groups were comparable in terms of patency and recurrence rates (*p*=0.777) as well as rates of epiphora (*p*=0.212).

**Conclusion:**

Both EX-DCR procedures were effective in the management of nasolacrimal duct obstruction. Our results suggest that EX-DCR augmented with 5-FU may represent a more feasible and cost-effective therapeutic option as compared to silicone tube placement in these patients.

## 1. Introduction

External dacryocystorhinostomy (EX-DCR) is the common surgical procedure to treat epiphora due to primary acquired nasolacrimal obstruction, with success rates usually greater than 90% [1, 2]. However, several factors may affect success rates, including the size of the bony ostium, membranous occlusion of the rhinostomy site, and recurrence surgery. Membranous failure secondary to soft tissue scarring at the rhinostomy site is considered to be the most common cause of primary EX-DCR failure [3].

A variety of methods can be used in DCR surgery to ensure controlled wound healing and prevent ostium closure. Polyethylene tubes, silicone sponges, silicone tubes, and absorbable gelatin sponges have all been utilized to improve surgical outcomes [2, 4–6]. The use of silicone tubing to facilitate repair of the nasolacrimal system was originally described by Gibbs, who first reported its use in the repair of damaged canaliculi in 1967 [7]. However, silicone tube intubation may also be associated with a plethora of complications, including foreign body reaction, reduced patient comfort, epistaxis, canaliculitis, fibrous reactions, and cheese-wiring [8].

Antimetabolite agents can be used to reduce scar formation and to improve functional outcomes after surgery. 5-Fluorouracil (5-FU) and mitomycin-C (MMC) are the most commonly used antimetabolites in ophthalmology. 5-FU inhibits fibroblast formation, as it blocks the enzyme thymidylate synthase involved in DNA synthesis. Tissue response to 5-FU is far more pronounced as compared to many other antimetabolites, with strong inhibitory effects on activated fibroblasts [9]. Mitomycin-C (MMC) is another potent antimetabolite agent, reducing collagen synthesis with marked effects on fibroblasts. MMC is also effective in wound healing, alleviating uncontrolled wound healing, and improving success rates in certain types of ocular surgery [10, 11]. Furthermore, 5-FU and MMC can be used to control conjunctival wound healing in surgery involving ocular surfaces as well as in oculoplastic surgery [12].

This study was carried out to compare anatomic and functional success rates in EX-DCR with adjunctive 5-FU or silicone intubation.

## 2. Materials and Methods

The research protocol was approved by the Institutional Ethics Committee. The study was waived from informed consent since clinical data did not include any personal identity information of patients.

In this retrospective study, medical records of patients undergoing an EX-DCR procedure between March 2018 and October 2019 at Sisli Hamidiye Etfal Training and Research Hospital were retrieved from the hospital database. In total, 80 EX-DCR procedures were performed in 72 patients with epiphora, due to primary acquired nasolacrimal obstruction. EX-DCR with adjunctive 5-FU was undertaken in 37 eyes of 32 patients (5-FU group), and EX-DCR with silicone intubation was performed in 43 eyes in 40 patients (control group). All procedures were performed by the same surgeon, and all patients underwent complete ophthalmological (including lacrimal syringing) and otorhinolaryngological examination before surgery. All patients with nasolacrimal duct blockage, as confirmed by syringing, were included. Patients with obstruction of the upper or lower canaliculi, eyelid and cornea anomalies, previous history of oculoplastic surgery, severe septal deviation, and turbinate hypertrophy were excluded.

Recent studies reported significantly better anatomic and functional outcomes and lower recurrence rates with EX-DCR with silicone intubation compared to EX-DCR without silicone intubation [13]. Some authors believe that silicone intubation can stimulate granulation tissue formation and lead to scarring with consequent relapse symptoms [13–16] while others believe that stent intubation may improve functional outcomes by maintaining the anastomotic lacrimal patency and the stability of the epithelium, and many surgeons routinely intubate silicone tubes during EX-DCR to improve the outcomes. However, numerous studies have reported that the long-term outcomes are not high especially in adults [17–20]. In our clinical practice, silicon intubation is routinely performed in EX-DCR procedures to achieve better surgical outcomes, in line with the published data.

As 5-FU is a powerful inhibitor on fibroblast proliferation, we used 5-FU as an alternative procedure to silicone intubation on patients who did not accept because of the potential complications and problems like foreign body sensation, nasal and conjunctival irritation, corneal abrasion, dislocation of the silicone tube, epistaxis, and cheese-wiring of the punctum that may arise over time.

### 2.1. Surgical Technique

All patients were operated under general and local regional anesthesia. A nasal pack soaked with lidocaine 2% with 1 : 100,000 epinephrine was inserted into the nose. After a *Z*-shaped skin incision 8–10 mm medial to the medial canthus and blunt dissection to the periosteum were made, the lacrimal fossa was exposed in its entirety. A nasal osteotomy of 15 × 15 mm was created over the lacrimal fossa with Kerrison bone punch. The lacrimal sac and nasal mucosa were cut in longitudinal U-shape anterior flaps. The smaller posterior flaps were excised. In the 5-FU group, a neurosurgical cotton was cut into a small triangular shaped pledgets and soaked with 5-FU at a concentration of 50 mg/ml, and four cotton pledgets were applied over the anterior flaps and osteotomy site for 2 minutes. In the control group, Crawford lacrimal intubation tubes (Visitec, UK) were inserted. After applying 5-FU and intubation in the respective groups, anterior mucosal flaps were sutured with two interrupted 6.0 Vicryl sutures. Upon completion of the mucosal anastomosis, the medial canthal ligament and orbicularis muscle were sutured with 6.0 Vicryl suture, and skin flaps with 6.0 polypropylene (Prolene) suture.

Patients were examined on postoperative days 1 and 15 and at 1, 6, and 12 months and after that, as needed. To evaluate the long-term results in both groups, subjective symptoms such as epiphora and objective findings such as patency of irrigation and recurrence rates were documented at 12-month follow-up after the operation. The success rate was evaluated by lacrimal patency to irrigation and relief of epiphora.

A comparison of continuous variables between two independent groups was performed using the Mann–Whitney *U* test and independent *t* test. Chi-square test was used to compare the success rate between the two groups. Alpha significance level was set at *p* < 0.05.

## 3. Results

A total of 80 DCR surgeries were performed in 72 patients. Eight patients underwent bilateral surgery. In EX-DCR plus 5-FU group (*n* = 37), there were 32 patients (22 female, 10 male), with a mean age of 59.8 ± 9.4 years (range 40–80 years) and a mean follow-up time of 18.70 ± 3.47 months (Table 1). Thirty-one of 37 eyes had a patent nasolacrimal duct on syringing, corresponding to a success rate of 83.8%, and postsurgical epiphora was observed in 6 of 37 eyes (16.2%) (Table 2). In the control group with silicone tube intubation (*n* = 43 eyes), there were 40 patients (28 female. 12 male), with a mean age of 57.0 ± 15.3 years (range 15–80 years) and a mean follow-up of 21.38 ± 7.76 months (Table 1). Thirty-seven of 43 eyes had a patent nasolacrimal duct on syringing with a success rate of 86.0%, and postsurgical epiphora was observed in 9 of 43 eyes (20.9%) (Table 2). In each group, recurrent cases who had epiphora and no patency were seen within 4 to 6 weeks after surgery. The recurrence rate was 16.2% in the 5-FU group and 20.9% in the control group. The two groups were comparable in terms of anatomic success rates (*p*=0.777 and *p*=0.212, respectively) as well as recurrence rates (Table 2).

Study groups were well matched for age, gender, and follow-up time. No local or systemic complications from the use of 5-FU were observed in the study.

## 4. Discussion

In this study, we observed no significant differences in lacrimal patency and recurrence rates as well as functional symptoms in patients undergoing EX-DCR either with 5-FU or silicone intubation.

Standard EX-DCR is a highly successful surgical procedure in primary acquired nasolacrimal duct obstruction and has been the procedure of choice since 1904 [21]. The advantages of EX-DCR include direct visualization of anatomy allowing accurate anastomosis between the lacrimal sac and the nasal mucosa [22]. Although endonasal DCR became popular in recent years because of its shorter operation time, absence of cutaneous scar, preserving medial canthal structures, allowing to be performed under local anaesthetic, outcome results have been disappointing when compared to the external route [23]. So, we chose the external technique for its higher success rate often reported to be more than 90% [24] and additional factors such as the surgeon's experience and operating costs. Also, we overcame scar problems by applying *Z*-plasty skin incision and none of the patients complained about the scar. Also, an assessment report of American Academy of Ophthalmology comparing endonasal and EX-DCR could not find significant differences between the two approaches [25].

The surgery has a very low failure rate of between 1% and 20% [26–29]. The main cause of surgical failure involves the development of obstruction at the rhinostomy site (ostium) [24]. Although opening the large ostium is an important determinant of surgical success, the accompanying fibroblastic response and scarring need to be controlled [30]. Until now, a variety of techniques and approaches including polyethylene tubes, silicone sponges, silicone tubes, and absorbable gelatin sponges have been used to achieve better surgical outcomes. Antimetabolite agents such as 5-FU and MMC have also been used to increase surgical success, owing to their ability to prevent fibroblast proliferation and scar formation [31]. 5-FU was first used in ophthalmic practice by Blumenkranz et al. in 1982 [32]. One of the first clinical trials on the use of 5-FU in trabeculectomy was performed by Heuer et al. [33]. Since then, it has been frequently used as an intraoperative or postoperative adjunct.

Only a small number of studies evaluated the effect of 5-FU on the outcome of EX-DCR surgery. In contrast with publications suggesting that 5-FU can prevent ostium obstruction, others failed to observe better results in terms of recurrence rates [25, 34, 35]. In Gonzalvo et al.'s study utilizing MMC in patients undergoing EX-DCR, larger ostial dimensions were achieved as documented by helical computed tomographic dacryocystography [36]. In our study, the use of 5-FU did not appear to have a significant impact on postoperative outcomes as compared to silicone intubation. On the other hand, Costa et al. found a higher rate of flap failure with 5-FU when compared to saline injection [37]. Furthermore, at 60 days of follow-up, surgical ostium was smaller in the 5-FU group than in the saline group; nevertheless, the final size of the surgical ostium was similar in the long term. On the other hand, Linberg et al. failed to observe any direct relationship between internal ostium size and surgical outcomes in patients undergoing EX-DCR [38]. Also, Piaton et al. and Bakri et al. emphasized that the use of 5-FU in patients undergoing laser DCR showed no superior effect on the surgical outcome [26, 39]. In the present study, 5-FU application to the nasal ostium resulted in non-inferior anatomical and functional success rates when compared with silicone tube intubation.

In a 2013 study by Cheng et al., endonasal DCR combined with an antimetabolite (MMC) was associated with higher success rates than controls, while DCR with silicone intubation or antimetabolite achieved comparable results [11]. This is similar to our findings, which indicate no difference between EX-DCR procedures carried out with 5-FU or silicone tube intubation.

In a recent review comparing antimetabolites (MMC and 5-FU) with control treatments in patients undergoing DCR, a slight superiority of these agents in terms of anatomical and functional outcomes was reported. In that review, it was stated that studies usually utilized MMC as the antimetabolite agent, and no studies examined the usefulness of 5-FU independently, although its use in patients with nasolacrimal duct obstruction was recommended [40]. Some of the studies included in that review also used silicone intubation and antimetabolites concurrently, which precludes an assessment of the effect of 5-FU or MMC alone on the outcomes. Since 5-FU and silicone intubation were not combined in our study groups, we believe that our results may be viewed as a contribution to the existing literature since they allow a comparison between antimetabolite agents alone and another adjunctive method.

In this study, we aimed to compare two different techniques as an adjunctive approach in patients undergoing EX-DCR. Although the success and failure rates associated with these two techniques were similar, 5-FU augmented EX-DCR allowed a shorter duration of surgery and eliminated tube-related complications postoperatively. Avoidance of the use of tube implantation may represent an advantage for surgeons without adequate expertise in EX-DCR. Also, 5-FU is more cost-effective compared to silicone intubation. Besides that silicone intubation is associated with longer surgery time, a steeper learning curve, difficulties of removal of the tube from the nasal cavity in patients with pathologies such as septal deviation, nasal polyps, and inferior concha deformity, as well as tissue edema and hemorrhage, it appears that adjunctive 5-FU usage in EX-DCR may represent a viable alternative to silicone intubation.

Some limitations of our study should be mentioned. Since this was not a large randomized controlled study with a longer follow-up, it was not possible to reach a more definitive conclusion regarding the long-term success rates with this approach. Also, we could not compare the ostium size using endonasal endoscopy. The timing of the application of 5-FU as well as its potential local side effects on the nasal mucosa is unknown. Furthermore, long-term studies involving a larger number of patients stratified according to preoperative symptom severity would shed more light on the effectiveness of the techniques discussed herein. Despite these limitations, to the best of our knowledge, this is the first study comparing anatomic and functional outcomes as well as recurrence rates with 5-FU versus silicone tube implantation in patients undergoing EX-DCR.

Both techniques used in EX-DCR are useful in the management of lacrimal duct obstruction. It is easier, quicker, and more cost-effective to apply 5-FU compared to implanting a silicone tube, and there is no statistically significant difference in the anatomic and functional success rates and recurrence rates between the two groups.

## Figures and Tables

**Table 1:** Demographic characteristics of the 5-FU and control groups.

		5-FU group (*n* = 37)	Control group (*n* = 43)	*p*
Age mean ± SD (min-max)		59.8 ± 9.4 (40–80)	57.0 ± 15.3 (15–80)	0.703^*∗*^
Gender *n* (%)	Male	10 (31.2)	12 (30.0)	0.990^†^
	Female	22 (68.7)	28 (70.0)	
Side (%)	Right/left	19 (51.4)/18 (48.6)	23 (53.5)/20 (46.5)	0.849^†^
Duration of follow-up (months) Mean ± SD (min-max)		18.70 ± 3.47	21.38 ± 7.76	0.055^*∗∗*^

^*∗*^Mann–Whitney *U* test; ^†^chi-square test; ^*∗∗*^independent *t* test.

**Table 2:** Lacrimal patency and recurrence rates of the groups.

	5-FU group	Control group	*p*
Lacrimal patency, *n* (%)	31 (83.8)	37 (86.0)	0.777
Epiphora (recurrence), *n* (%)	6 (16.2)	9 (20.9)	0.212

## Data Availability

The datasets used and/or analyzed during the current study are available from the corresponding author upon reasonable request.

## Abbreviations

EX-DCR: External dacryocystorhinostomy
5-FU: 5-Fluorouracil
MMC: Mitomycin-C.
